# Left Ventricular Longitudinal Function Assessed by Speckle Tracking Ultrasound from a Single Apical Imaging Plane

**DOI:** 10.1155/2012/361824

**Published:** 2012-04-24

**Authors:** Thomas Bagger, Erik Sloth, Carl-Johan Jakobsen

**Affiliations:** Department of Anaesthesia and Intensive Care, Aarhus University Hospital, Skejby, 8200 Aarhus N, Denmark

## Abstract

*Background*. Transthoracic ultrasonography of the heart is valuable in monitoring and treatment of critically ill patients. Speckle tracking ultrasound (STU) has proven valid in estimating left ventricular systolic deformation. The aims of the study were to compare conventional and automated STU and to determine whether left ventricular systolic deformation could be estimated from one single imaging plane. *Methods*. 2D-echocardiography cine-loops were obtained from 20 patients for off-line speckle tracking analysis, consisting of manually tracing of the endocardial border (conventional method) or automatically drawn boundaries (automated method). *Results*. We found a bias of 0,6 (95% CI −2.2−3.3) for global peak systolic strain comparing the automated and the conventional method. Comparing global peak systolic strain of apical 4-chamber cine-loops with averaged Global Peak Strain obtained from apical 4, 2 and long axis cine-loops, showed a bias of 0.1 (95% CI −3.9−4.0). The agreement between subcostal 4-chamber and apical 4-chamber global peak systolic strain was 4.4 (95% CI −3.7−12.5). *Conclusion*. We found good agreement between the conventional and the automated method. STU applied to single apical 4-chamber cine-loops is in excellent agreement with overall averaged global peak systolic strain, while subcostal 4-chamber cine-loops proved less compliant with speckle tracking ultrasound.

## 1. Introduction

Bedside transthoracic ultrasound protocols have won wide spread use for monitoring and guiding treatment of the critically ill patients [1–7]. Evaluation of left ventricular systolic function is a key element in focused protocols [1, 2] as well as standard echocardiography [8]. Visual estimation (eyeballing) and wall motion index (WMI), Simpsons Biplane method, and Doppler tissue imaging are used for quantification of left ventricular systolic function, but they are either subjective, dependent upon operator experience, or time consuming [9–17]. Speckle Tracking Ultrasound (STU) is a novel method allowing assessment of both regional and global left ventricular function [18–21] in dedicated semiautomatic software, thereby making this method fast and potentially available for online real-time analysis in the critical setting where time is crucial.

Two different STU algorithms are available in the Echopac software (GE Healthcare, Horten Norway). One is conventional quantitative strain analysis (Q-analysis) in which manual tracing of the endocardial border is necessary and secondly a less time-consuming automated function imaging (AFI) where the endocardial borders are automatically traced. Quite often the image quality from the AP4C view is poor in critically ill patients for different reasons: positive pressure ventilation, catheters, wires, surgical dressing, and posture among the most important. Therefore we set out to examine whether GLPS could be estimated from a single subcostal view.

Thus the purpose of this study was to compare the two methods and secondly to evaluate whether left ventricular deformation could be estimated from a single imaging plane by means of STU.

## 2. Materials and Methods

### 2.1. Patients

The study was approved by the Danish Data Protection Agency (no. 2011-41-6010) with a waiver of informed consent. Bedside ultrasonography is a standard component of clinical care at the site of investigation. 20 patients (13 women and 7 men), mean age 66 (range 25–90) years, who previously underwent comprehensive conventional 2-dimensional echocardiography were studied. Two patients underwent coronary artery bypass grafting and 15 aortic valve replacement surgery due to either aortic valve stenosis (13) or aortic valve regurgitation (2), while three patients had no known cardiac disease.

### 2.2. Data Acquisition and Analysis

Transthoracic ultrasonography images were obtained by an experienced ultrasonographer using General Electric Vivid 9 system equipped with an M4S probe (frequency range: 1.5–4.0 MHz). Grey-scale 2-dimensional (2D) ECG-triggered, apical 2-chamber (AP2C), apical long axis (APLAX), apical 4-chamber (AP4C), and subcostal 4-chamber (SU4C) cine-loops were recorded with frame rates ranging from 42 to 70 fps for offline analysis.

From each view one cardiac cycle was selected for analysis of 2D strain with the two methods. First is *Q-analysis* (*conventional method)*: the myocardial wall of the left ventricle was outlined by manually applying successive points along the endocardial border followed by automated tracing of the epicardial border and thus defining a region of interest (ROI). *AFI (automated function imaging) method*: two points were applied on each side of the mitral valve and a third point at the apex of the left ventricle followed by automated tracing of endocardial and epicardial borders defining ROI. Manual readjustment of endocardial tracing and ROI were performed in both methods in order to achieve optimal alignment if necessary. Aortic valve closure marked end systole and was defined automatically in the apical long axis view at the end of the T-wave of the corresponding electrocardiographic tracing and used as a reference for the subcostal, four- and two-chamber views. Time of aortic valve closure was also visually confirmed and adjusted if necessary.

**Figure 1:**
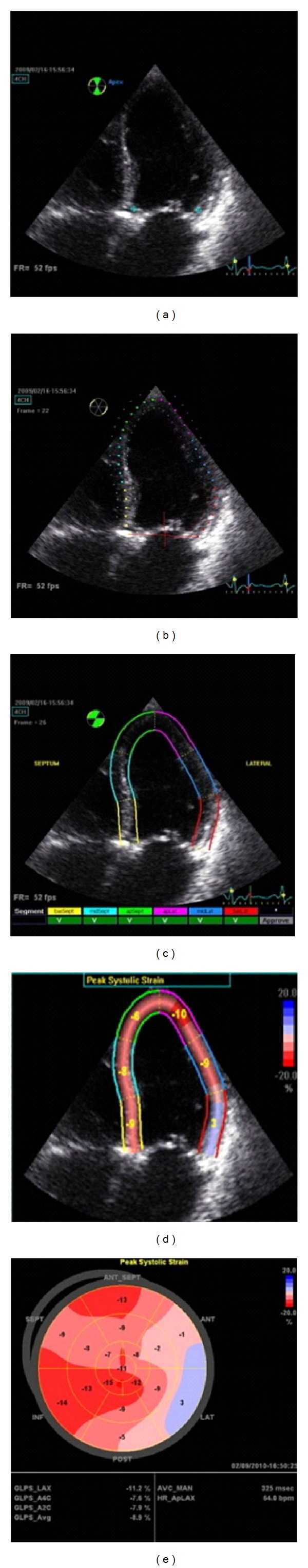
Steps involved in the sequence of Automated Functional Imaging (AFI) analysis and conventional analysis using the apical four chamber view (AP4C). (An example, see also text.) (a) Two points have been applied on both sides of the Mitral valve. The apical point has yet to be placed. (b) Myocardial wall of the left ventricle has been outlined defining a region of interest (ROI), consisting of 3 concentric lines delineating the endocardial and epicardial borders and a midmyocardial layer and end systole is automatically marked by aortic valve closure. (c) ROI is divided into 6 segments and good alignment is confirmed within each segment. (d) Peak systolic strain values of each segment have been calculated by the computer algorithm. (e) Bulls Eye plot presenting segmental peak systolic strain values (17 segment model) and global peak systolic strain values of each view (GLPS LAX: apical long axis; GLPS 4C: apical four-chamber; GLPS 2C: apical 2 chamber) and an overall averaged peak systolic strain value average GLPS.

In both methods, the region of interest outlining the entire left ventricular wall was divided into 6 segments. A computer algorithm calculated peak systolic strain values within each segment together with global peak systolic strain (GLPS) from each view and lastly overall averaged global peak systolic strain (aGLPS) of the AP4C, AP2C, and APLAX views (Figure 1). Cine-loop analysis of the SU4C was conducted with the same algorithm used for analysis of AP4C views. All analysis was done by two independent observers to estimate interobserver variability.

### 2.3. Statistical Analysis

Bland-Altman analysis was used to calculate the bias and limits of agreement between corresponding measurements. Analysis was done with MedCalc software version 11.5.1 (Mariakerke, Belgium).

## 3. Results

**Figure 2:**
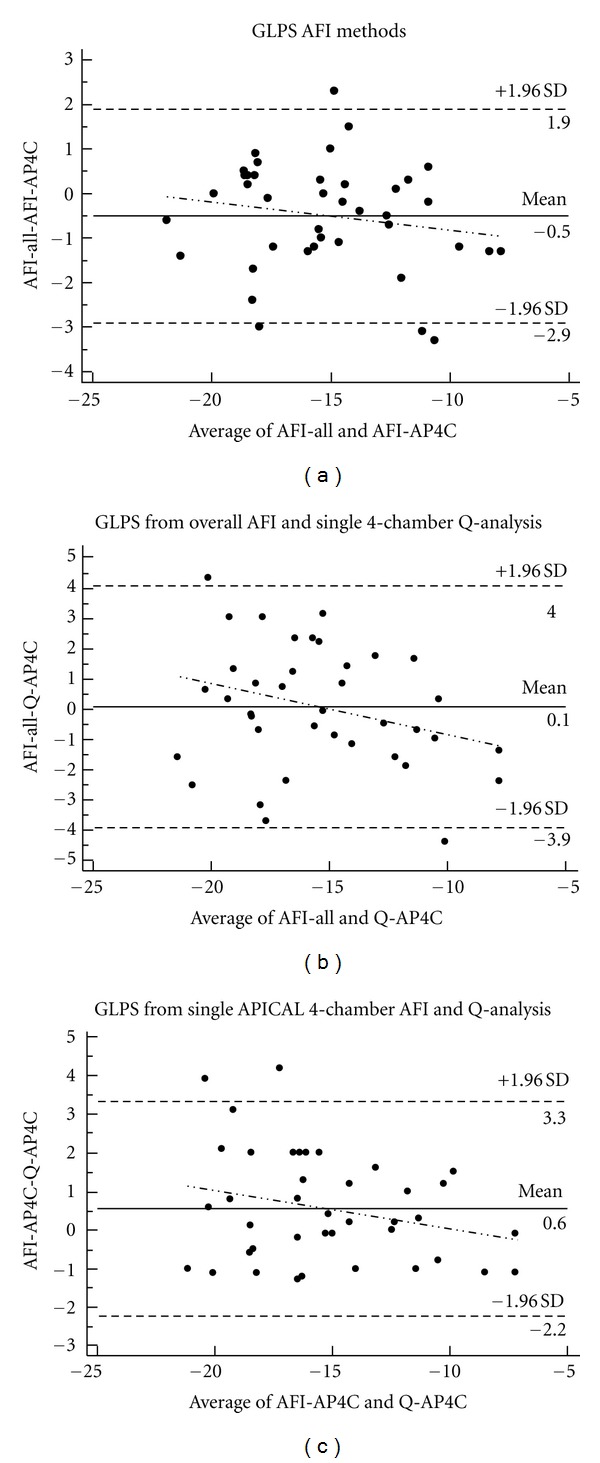
Bland Altman plot with mean difference and limits of agreement between overall averaged global peak systolic strain (aGLPS) of the AP4C, AP2C, and APLAX views using AFI method (AFI-All) compared to single apical 4-chamber GLPS using AFI method (AFI-AP4C) (a), AFI-all compared to single apical 4-chamber view using conventional Q-analysis (Q-AP4C) (b), and GLPS comparing AFI of an apical single 4-chamber view (AFI-AP4C) and Q-analysis of an apical single 4-chamber view (Q-AP4C) (c).

80 cine-loops were analyzed representing a total of 480 segments of which none were excluded. Bland Altman analysis of aGLPS (AFI-all) and GLPS from a single apical four-chamber view (AFI-AP4C) showed a mean difference of −0.5 with 95% confidence limits (95% CI) between −2.9 and 1.9 (Figure 2).

Bland Altman analysis of aGLPS using the AFI method (AFI-All) against GLPS obtained from conventional Q-analysis of single apical 4-chamber view (Q-AP4C) showed a mean difference of 0.1 (95% CI −3.9–4.0), while comparison of conventional Q-analysis of a single apical 4-chamber and AFI analysis of a single apical 4-chamber view showed a mean difference of 0.6 (95% CI −2.2–3.3) (Figure 2).

**Figure 3:**
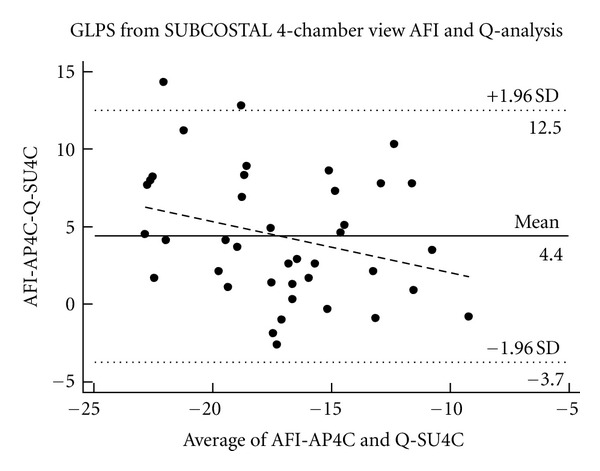
Bland Altman plot with mean difference and limits of agreement between Q-analysis of global peak systolic strain from the subcostal 4-chamber view (Q-SU4C) view compared to AFI global peak systolic strain (AFI-AP4C).

The mean difference comparing conventional Q-analysis of GLPS (Q-SU4C) and GLPS using the AFI method (AFI-AP4C) from single subcostal 4-chamber view was 4.4 (95%  −3.7–12.5) (Figure 3).

**Figure 4:**
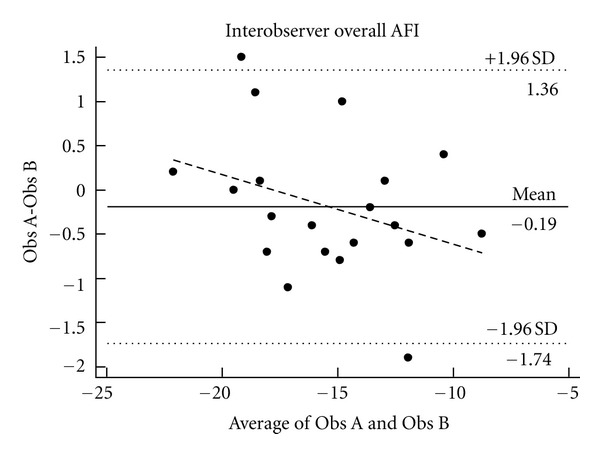
Bland Altman plots comparing observer A with observer B using AFI.

The agreement between the two observers using the AFI showed a mean difference of −0.19 with 95% limits of −1.74–1.36 (Figure 4). For the conventional analysis of apical 4-chamber view, the mean differences, was −0.5 (95% CI −4.2–3.1), while the conventional analysis of subcostal 4-chamber view was −1.5 (95% CI 8.0–11.0).

## 4. Discussion

We found good agreement between average global peak systolic strain obtained by the conventional method and the AFI method, although the two methods basically utilize the same algorithm calculating strain and the fact that the two methods are interchangeable is extremely relevant in critical care because the time consumption is very different for the two methods.

Our study showed that left ventricular systolic deformation could be estimated using the AFI method from one cine-loop of a single AP4C view. This has also tremendous critical care application since the apical two- and long-axis view are both time demanding and difficult to achieve in the critical scenario. This finding also applies to most of the focussed echo protocols which disregard the apical 2- and long-axis view [1–3]. Recently we have shown that the AP4C view can be achieved even with the patient in the sitting position [4]. The fact that AFI is already available on several high-end ultrasound machines makes these results even more attractive.

**Figure 5:**
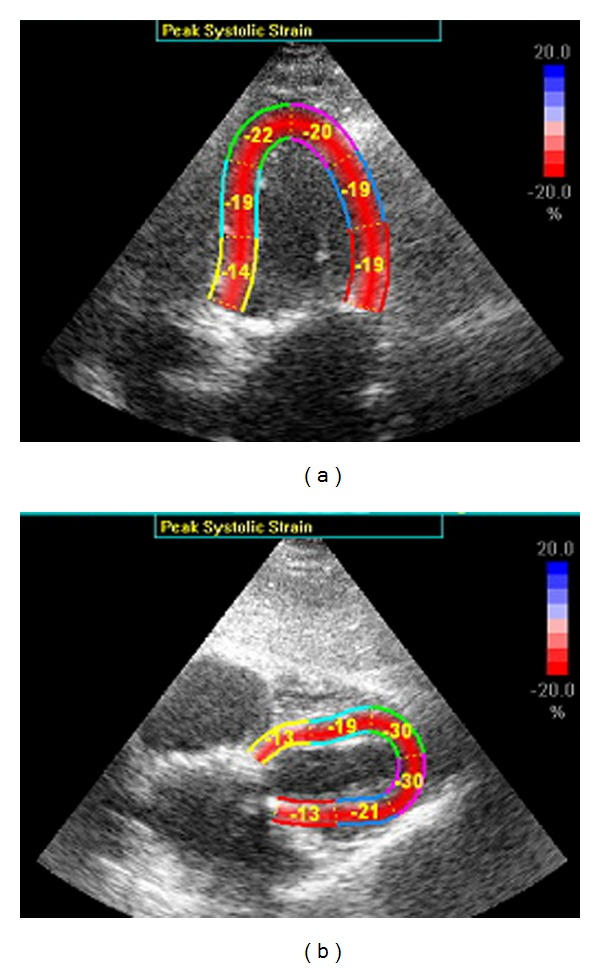
Apical (a) and subcostal (b) four-chamber view from the same patient. Segmental peak systolic strain values are indicated. Apex of the heart can be difficult to recognize in the subcostal view and peak systolic strain values appear to be significantly higher at the apex of the subcostal view than the apical view. See text for further explanation.

In critical care, most patients are placed supine for different reasons making the subcostal four-chamber view the a priori best choice. Unfortunately our results showed that GLPS from Sub4C significantly overestimates GLPS from both AP4C and aGLPS. First of all it was difficult to obtain GLPS from SU4C using the AFI method because this required substantial correction of ROI making this analysis no different from the conventional method and thereby eliminating the time advantage of the AFI method. Secondly we noticed that overestimation primarily was presented at apical segments (Figure 5). One reason could be that interposition of inflated lung often blurs the apex of the heart in the subcostal view. Another reason could be that the algorithm used to calculate strain from AP4C is not appropriate for the Sub4C, because the images are tilted approximately 90 degrees from having the axis of the heart at the center of the ultrasound beam to a lateral position. Sivesgaard et al. [22] demonstrated that STU is independent of insonation angle, but reliability was dependent upon speckle tracking number (STN). STN describes the relation between displacement, frame rate, and sector depth, and beyond a certain value peak strain cannot be reliably measured. One could speculate that the subcostal view represents STN values beyond the critical value. The subcostal view requires greater sector depth compared to apical views and this could be the reason why STU applied to cine-loops of the subcostal view results in substantial overestimation of systolic peak strain values. Future software optimisation might improve the assessment of STU from SU4C.

Using a single view is limited by not describing regional differences in myocardial motion and this must be taken into consideration. Reduced myocardial motion will not be detected in the posterior or anterior wall leading to an overestimation of left ventricular systolic function. Conversely underestimation will likely take place if reduced myocardial motion occurs in the septal or lateral wall.

We found good agreement between the experienced and the novel observer using either conventional Q-analysis or AFI. This indicates that emergency physicians with limited experience can perform analysis, but results are dependent upon good quality ultrasonographic images, which can be difficult for the inexperienced to obtain. Studies have shown that the task of performing focused bedside transthoracic ultrasonography can be learned fast even with little or no previous ultrasonography experience [5, 20].

## 5. Conclusion

This study shows that global peak systolic strain based on AFI is in good agreement with conventional Q-analysis for the single apical 4 chamber. STU applied to the subcostal four-chamber view cannot replace the apical four-chamber view. AFI method has the potential to become the method of choice in clinical settings, because it is fast and more objective opposed to visual eyeballing and can be performed with even limited experience of ultrasonography.
